# Decoding Lupus Diagnosis, Pathogenesis and Therapy: From Systemic Autoimmunity to Renal Damage

**DOI:** 10.3390/diagnostics16142170

**Published:** 2026-07-11

**Authors:** Giuseppe Stefano Netti, Dario Troise, Barbara Infante, Michele Rossini, Valentina Camporeale, Federica De Luca, Giorgia Leccese, Federica Galloso, Roberto Cuttano, Francesca Sanguedolce, Loreto Gesualdo, Giovanni Stallone, Elena Ranieri

**Affiliations:** 1 Unit of Clinical Pathology, Department of Medical and Surgical Sciences, University of Foggia—University Hospital “Policlinico Riuniti”, Viale Luigi Pinto, 71122 Foggia, Italy; valentina.camporeale@unifg.it (V.C.); federica.deluca@unifg.it (F.D.L.); giorgia_leccese.555553@unifg.it (G.L.); federica_galloso.563213@unifg.it (F.G.); roberto.cuttano@unifg.it (R.C.); elena.ranieri@unifg.it (E.R.); 2 Center for Research and Innovation in Medicine (CREATE), Department of Medical and Surgical Sciences, University of Foggia—University Hospital “Policlinico Riuniti”, Viale Luigi Pinto, 71122 Foggia, Italy; dario.troise@unifg.it (D.T.); barbara.infante@unifg.it (B.I.); giovanni.stallone@unifg.it (G.S.); 3 Unit of Nephrology, Dialysis and Transplantation, Advanced Research Center on Kidney Aging (A.R.K.A.), Department of Medical and Surgical Sciences, University of Foggia—University Hospital “Policlinico Riuniti”, Viale Luigi Pinto, 71122 Foggia, Italy; 4 Unit of Nephrology, Dialysis and Transplantation, Department of Precision and Regenerative Medicine and Ionian Area (DiMePRe-J), University of Bari “Aldo Moro”, Policlinico, Piazza Giulio Cesare 11, 70124 Bari, Italy; michele.rossini@uniba.it (M.R.); loreto.gesualdo@uniba.it (L.G.); 5 Cancer Cell Signalling Unit, Fondazione IRCCS “Casa Sollievo della Sofferenza”, 71043 San Giovanni Rotondo, Italy; francesca.sanguedolce@unifg.it; 6 Unit of Pathology, Department of Clinical and Experimental Medicine, University of Foggia—University Hospital “Policlinico Riuniti”, Viale Luigi Pinto, 71122 Foggia, Italy

**Keywords:** systemic lupus erythematosus, lupus nephritis, innate immunity, adaptive immunity, targeted therapies

## Abstract

Systemic lupus erythematosus (SLE) is a chronic autoimmune disorder characterized by the loss of self-tolerance to nuclear and cytoplasmic antigens, triggering immune activation and tissue inflammation. Lupus nephritis (LN) is a major determinant of disease-related morbidity, disability, chronic kidney disease progression, kidney failure, and mortality in SLE, affecting approximately 30% of patients at diagnosis and up to 50–60% within the first decade. This review examines the disease’s pathogenic mechanisms, emphasizing the innate immune system’s role in the loss of self-tolerance and subsequent activation of the adaptive immune response. Mechanisms include dysregulated cell death pathways, impaired clearance of nucleic acid-containing debris and immune complexes, and involvement of antigen-presenting cells and other innate immune cells. These processes lead to the clonal expansion of autoreactive lymphocytes, generating effector T cells, memory B cells, and plasma cells that produce autoantibodies, resulting in renal injury. The review further explores the immunological processes driving kidney damage, beginning with autoantibody binding and immune complex deposition, followed by complement-mediated microvascular injury, kidney stromal cell activation, and leukocyte recruitment. Lastly, it discusses LN treatment strategies, from traditional to novel targeted therapies, with a focus on their systemic immunologic impacts and the protection of podocytes.

## 1. Introduction

Systemic lupus erythematosus (SLE) has been defined as a chronic autoimmune disease wherein the human immune system mistakenly targets nuclear and cytoplasmic self-antigens, driving inflammation across multiple organs.

This aberrant immune response leads to the production of various autoantibodies, forming immune complexes that contribute to tissue inflammation. This breakdown in self-tolerance typically affects genetically susceptible individuals exposed to environmental triggers. Extensive research, including genome-wide association studies, has revealed numerous genetic risk factors and HLA associations underlying this loss of self-tolerance in SLE [1,2,3].

Kidney disease arising as a consequence of SLE is termed lupus nephritis (LN). LN is one of the strongest determinants of adverse long-term outcomes in SLE, contributing to disease-related morbidity, disability, chronic kidney disease progression, kidney failure, and increased mortality compared with the general population. Various pathogenic pathways contribute to the development of both SLE and LN. Therefore, understanding the spectrum of these mechanisms holds promise for tailoring personalized treatment approaches. Nonetheless, the accumulation of a vast amount of data on SLE and LN over recent decades has resulted in complexity. This review aims to provide an integrated and clinically oriented overview of SLE and lupus nephritis, linking diagnostic reasoning, immune-pathogenic mechanisms, renal tissue injury, and therapeutic decision-making. Rather than presenting SLE and LN as separate entities, we follow the continuum from loss of immune tolerance to systemic autoimmunity, intrarenal immune activation, histological injury patterns, and treatment strategies tailored to disease phenotype and renal involvement.

## 2. Diagnosis of Systemic Lupus Erythematosus

Diagnosing systemic lupus erythematosus (SLE) remains challenging due to its heterogeneous and often subtle early manifestations. The diagnostic approach aims to identify clinical pictures suggestive of SLE and to properly integrate clinical features with serological data in order to reduce diagnostic ambiguity.

SLE commonly begins with constitutional, musculoskeletal, or mucocutaneous symptoms. Fatigue—although not included in classification criteria due to its nonspecific nature—is highly prevalent and should prompt clinicians to consider additional organ involvement, particularly when it co-occurs with rashes, joint symptoms, or other suggestive features. Importantly, some patients initially present with single-organ involvement such as lupus nephritis, highlighting the need for a comprehensive history covering symptom chronology, systemic review, and family history of autoimmune diseases.

Musculoskeletal manifestations are among the most frequent. Up to 90% of patients experience musculoskeletal symptoms during the disease course. Inflammatory arthralgia may be migratory, transient, or persistent, and typically affects small and medium joints, especially the hands, wrists, and knees. Imaging reveals synovitis, tendon involvement, and occasionally erosive disease, despite the traditional teaching that SLE is non-erosive. The classic Jaccoud arthropathy is now rare, occurring in fewer than 5% of patients [4].

Mucocutaneous disease is diverse and contributes significantly to early diagnosis. Overall, cutaneous or mucosal manifestations occur in the majority of patients during the disease course. Acute cutaneous lupus may present as the classic malar (‘butterfly’) rash, reported in approximately 30–50% of patients, while photosensitivity occurs in about 40–70%. Oral or nasal ulcers are observed in approximately 20–40%, non-scarring alopecia in 20–40%, discoid lesions in 5–20%, and subacute cutaneous lupus in a smaller but clinically relevant subset. Because these dermatologic manifestations vary widely in depth, morphology, distribution, and chronicity, clinical recognition remains crucial [4].

A major source of patient frustration, as noted in the literature, is the difficulty in obtaining a definitive diagnosis [5]. Although classification criteria are primarily intended for research, they serve as an important framework for clinicians. The 2019 EULAR/ACR criteria require a positive ANA (≥1:80) as an entry criterion, reflecting consensus that ANA positivity is a fundamental immunologic hallmark of SLE [6,7]. The criteria allocate weighted points across clinical and immunological domains; a total score of ≥10 points, with at least one clinical criterion, supports SLE classification. Only the highest-weighted item per domain counts, underscoring that the criteria reward breadth across systems rather than clustering within a single domain. A summary of key criteria and weightings towards SLE classification is shown in Table 1.

Indirect immunofluorescence on HEp-2 cells provides information not only on ANA titer but also on the intracellular distribution of the recognized antigens. According to the International Consensus on ANA Patterns, the homogeneous nuclear pattern (AC-1), characterized by diffuse staining of the interphase nucleus and condensed mitotic chromatin, is commonly associated with antibodies against double-stranded DNA, histones, and nucleosomes and is frequently observed in SLE. Nuclear speckled patterns, including fine and coarse speckled patterns (AC-4 and AC-5), may reflect reactivity against Sm, U1-RNP, Ro/SSA, or La/SSB antigens and are also common in SLE. Cytoplasmic patterns may occasionally indicate antibodies against ribosomal P proteins or other cytoplasmic antigens relevant to the lupus autoantibody profile. By contrast, isolated nucleolar or centromere patterns are less typical of SLE and should prompt consideration of systemic sclerosis or an overlap connective-tissue disease. The dense fine speckled pattern (AC-2), particularly when occurring in isolation and without disease-specific autoantibodies, is more frequently encountered in otherwise healthy individuals and may reduce the likelihood of a systemic autoimmune rheumatic disease. Nevertheless, fluorescence patterns are not disease-specific and should always be interpreted together with antibody specificity, titer, clinical manifestations, and other laboratory findings [8,9].

Although ANA positivity on HEp-2 cells is highly sensitive and represents the mandatory entry criterion for the 2019 EULAR/ACR classification criteria, rare ANA-negative SLE cases have been reported in clinical series. These cases should be interpreted cautiously, because apparent ANA negativity may reflect differences in assay substrate, laboratory methodology, antibody cut-off, immunosuppressive treatment before testing, or predominant autoantibody specificities not optimally detected by standard ANA screening. Some ANA-negative patients display anti-Ro/SSA antibodies, hypocomplementemia, antiphospholipid antibodies, or biopsy-proven immune-complex organ disease, including lupus nephritis. Pathogenetically, these cases support the concept that SLE may occasionally be driven by immune-complex formation, complement activation, and non-classical autoantibody profiles even when conventional ANA testing is negative. Therefore, ANA negativity strongly argues against SLE, but it should not completely exclude the diagnosis when there is compelling multisystem involvement and supportive immunological or histological evidence.

Regarding immunology, highly specific antibodies such as anti-Sm and anti-dsDNA strongly support the diagnosis. Other autoantibodies (anti-Ro/SSA, anti-La/SSB, anti-RNP, antiphospholipid antibodies) are supportive but less specific, requiring careful differential diagnosis of overlapping autoimmune conditions. This reinforces the importance of antibody profiles in both diagnosis and attribution (Table 2).

Assessment of disease activity is distinct from diagnosis but is essential once SLE is suspected or established. Validated activity indices, including SLEDAI-2K, SELENA-SLEDAI, BILAG, ECLAM, SLAM, and physician global assessment, provide standardized measures of inflammatory disease burden and are widely used in clinical trials and longitudinal cohorts. In clinical practice, these instruments help distinguish active inflammatory disease from irreversible damage or non-inflammatory symptoms, define flares, monitor response to therapy, and support treat-to-target strategies. This distinction is particularly relevant in patients with fatigue, pain, or fibromyalgia-like manifestations, in whom escalation of immunosuppression should be avoided unless objective inflammatory activity is present.

Another diagnostic complexity arises from early or partial disease presentations—variously termed “early lupus,” “incomplete lupus,” or “subcriteria lupus.” Most of such patients do not progress to definitive SLE, though 5–10% subsequently develop full disease or evolve into another connective-tissue disorder such as Sjögren’s syndrome or systemic sclerosis [10,11,12].

Careful monitoring is recommended, as risk factors for progression include young age at onset, serositis, anti-dsDNA positivity, or a high interferon gene signature. For persistent but unclassifiable symptoms lasting three or more years, the diagnosis of undifferentiated connective-tissue disease (UCTD) may be appropriate [13].

SLE also shares symptoms with common mimicking conditions. Fibromyalgia is especially challenging, as pain, fatigue, and positive ANA tests often lead to misattribution. Studies show substantial coexistence of fibromyalgia with SLE, prompting the conceptual model distinguishing type 1 (inflammatory) from type 2 (non-inflammatory, fibromyalgia-like) symptoms to guide more nuanced discussions with patients and to avoid unnecessary escalation of immunosuppression [14,15,16].

Ultimately, early referral to rheumatology is emphasized as essential. A structured diagnostic approach encourages clinicians to synthesize multisystem findings, weigh alternative diagnoses, and appropriately apply the EULAR/ACR criteria to arrive at more accurate and timely SLE diagnoses.

## 3. Recent Insights into the Pathogenesis of Systemic Lupus Erythematosus

Current models of SLE pathogenesis support a multistep process in which genetic susceptibility, environmental exposures, defective clearance of nuclear material, and dysregulated innate and adaptive immune responses converge to break immune tolerance. For clarity, the pathogenic framework can be organized into four interconnected axes: (i) genetic and epigenetic susceptibility affecting immune regulation and nucleic-acid sensing; (ii) abnormal cell death and defective efferocytosis, leading to persistence of immunogenic nuclear debris; (iii) innate immune activation, particularly through Toll-like receptors, cytosolic nucleic-acid sensors, plasmacytoid dendritic cells, neutrophils, complement, and type I interferon pathways; (iv) adaptive immune amplification, characterized by autoreactive T- and B-cell activation, germinal-center dysregulation, plasma-cell persistence, and autoantibody production [17].

Each of these axes is influenced by partially distinct but interconnected genetic programs. Variants affecting nucleic-acid sensing and type I interferon signaling, including IRF5, IRF7, STAT4, TYK2, IFIH1, and TREX1, may lower the activation threshold of antiviral-like immune responses to endogenous nucleic acids. Genes involved in the clearance of apoptotic debris and immune complexes, such as C1QA, C1QB, C1QC, C2, C4A/C4B, and DNASE1L3, contribute to the persistence of extracellular chromatin and nucleosome-containing material when defective. A third group of genes, including HLA-DR/DQ, PTPN22, BLK, BANK1, TNFAIP3, and TNFSF4, modulates antigen presentation, lymphocyte activation, and peripheral tolerance checkpoints. Finally, genes regulating immune-complex handling and inflammatory tissue injury, such as FCGR2A, FCGR3A, and ITGAM, influence Fc receptor signaling, leukocyte recruitment, and organ-level inflammation. Thus, genetic susceptibility does not act as a single upstream event but shapes the intensity and persistence of multiple immunological pathways that converge in SLE and LN [17].

**Figure 1:**
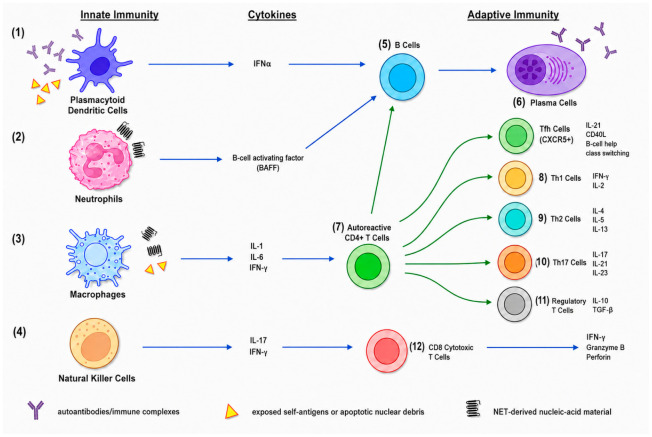
Immunological drivers of systemic lupus erythematosus: sequential activation of innate and adaptive immunity. In genetically susceptible individuals, defective clearance of apoptotic and NET-derived nuclear material promotes activation of plasmacytoid dendritic cells, macrophages, neutrophils, and other antigen-presenting cells. Nucleic-acid sensing through Toll-like receptors and cytosolic sensors induces type I interferons, BAFF, IL-6, TNF-α, and other inflammatory mediators. These signals activate autoreactive CD4+ T cells and promote their differentiation into Th1, Th2, Th17, T follicular helper, and regulatory T-cell subsets. Th1 cells amplify macrophage activation and inflammatory tissue injury; Th2 cells provide B-cell help and support antibody production; Th17 cells sustain IL-17-mediated inflammation; Tfh cells drive germinal-center B-cell activation, class switching, and plasma-cell differentiation; and defective Treg function fails to restrain autoreactive immune responses. The final result is B-cell expansion, plasmablast and plasma-cell persistence, autoantibody production, immune-complex formation, and tissue inflammation.

A central feature is the “interferon signature,” denoting the upregulation of interferon-stimulated genes, which contributes to disease onset and persistence [18]. Elevated expression of STAT1 and STAT2 in B cells and monocytes further underscores the role of type I interferon dysregulation [19]. Beyond interferons, other cytokines and costimulatory molecules are implicated in disease progression and represent potential therapeutic targets [17]. The complexity of these immune pathways reflects the multifactorial nature of SLE (Figure 1). Advances in understanding these mechanisms have enabled the identification of novel therapeutic targets and support the development of personalized treatment strategies [17,20]. Targeting multiple immune pathways may offer improved disease control and long-term remission.

From a mechanistic perspective, SLE can therefore be understood as a chronological and self-amplifying cascade. Genetically susceptible individuals exposed to environmental triggers develop inefficient clearance of apoptotic and NET-derived nuclear material. This material is recognized by innate immune sensors and promotes type I interferon production, complement activation, and antigen-presenting cell maturation. These innate events subsequently lower the threshold for autoreactive T- and B-cell activation, promote germinal-center abnormalities, sustain plasma-cell differentiation, and lead to the production of autoantibodies. Once immune complexes deposit in tissues, particularly in the kidney, complement activation, Fc receptor engagement, resident renal-cell activation, and leukocyte recruitment translate systemic autoimmunity into organ damage.

The initial break in tolerance occurs when normally hidden or rapidly cleared nuclear antigens become persistently available to the immune system in an inflammatory context. Apoptotic blebs, NET-derived chromatin, nucleosomes, DNA, RNA, histones, and ribonucleoproteins may undergo oxidative or other post-translational modifications that increase their immunogenicity. In genetically susceptible individuals, defective clearance and excessive nucleic-acid sensing allow these self-components to be taken up by dendritic cells, macrophages, and autoreactive B cells. Antigen presentation together with type I IFN, BAFF, IL-6, IL-21, and costimulatory signals permits survival and expansion of autoreactive lymphocyte clones that would otherwise remain deleted, anergic, or suppressed.

## 4. Innate Immunity and the Breakdown of Self-Tolerance in SLE

A defining feature of systemic lupus erythematosus (SLE) is the loss of immune tolerance to nuclear self-antigens, resulting in the production of antinuclear antibodies. This breakdown is closely linked to innate immune dysregulation, particularly involving cell death pathways and impaired clearance mechanisms [21,22].

Innate immunity acts as the first defense against nucleic acid-containing particles from pathogens [15]. To avoid inappropriate activation against endogenous nuclear material, the body relies on physiological systems—such as the complement cascade and circulating DNases—to eliminate chromatin debris and apoptotic cells [23]. Genetic predispositions and environmental triggers (e.g., smoking, UV exposure, infections) can disrupt these mechanisms, leading to accumulation of immunogenic debris and immune complexes that activate antigen-presenting cells (APCs) [2].

Efferocytosis is the physiological process by which apoptotic cells are recognized, engulfed, and degraded by professional and non-professional phagocytes before loss of membrane integrity occurs. The cells involved in this process, often referred to as efferocytes, include macrophages, dendritic cells, monocytes, and, in some tissues, epithelial or stromal cells. Efficient efferocytosis prevents secondary necrosis and limits extracellular exposure of nuclear antigens. In SLE, defective efferocytosis may result from impaired complement opsonization, altered bridging molecules, reduced DNase activity, or intrinsic macrophage dysfunction. Consequently, apoptotic blebs, nucleosomes, chromatin fragments, and nucleic-acid-containing immune complexes persist in the extracellular space, where they can activate Toll-like receptors and cytosolic nucleic-acid sensors in antigen-presenting cells. This provides a direct mechanistic link between innate immune dysfunction, loss of tolerance, type I interferon production, and autoantibody generation.

Epigenetic modifications also contribute to autoimmunity. Hypomethylation of DNA and RNA at specific loci may enhance the immunogenicity of nuclear material, promoting Toll-like receptor (TLR) activation [24,25]. Events such as trauma or sunburn can increase the release of immunogenic chromatin, triggering SLE flares [26]. Moreover, dysregulated cell death pathways—including apoptosis, necroptosis, and pyroptosis—are frequently observed in SLE patients [27]. These processes compromise membrane integrity and release modified nuclear antigens, which are more likely to be recognized by APCs such as plasmacytoid dendritic cells (pDCs), B cells, monocytes, and macrophages [23,26,28].

Nucleic acid-containing immune complexes are internalized by APCs through Fc receptors that bind the Fc portion of antibodies, while the nucleic-acid cargo subsequently activates endosomal sensors such as TLR3, TLR7, and TLR9 [29]. This leads to transcription of type I interferons (IFN-I) and pro-inflammatory cytokines including IL-6, IL-12, TNF-α, and BAFF [23]. These signals enhance autoantigen presentation and promote T-cell activation.

IFN-I, primarily IFN-α and IFN-β, is produced in large quantities by pDCs in response to viral-like stimuli. IFN-I and IFN-II (IFN-γ) share overlapping gene regulatory functions. Binding of IFN-I to its receptor initiates the “IFN signature,” activating both innate and adaptive immunity [30]. Although IFN-I levels are typically low, their biological effects are evident through the expression of IFN-stimulated genes. IFN-I enhances NK cell cytotoxicity, macrophage activation, dendritic-cell maturation, and T-cell priming [31,32].

Type I IFN promotes B-cell differentiation both directly and indirectly. It enhances dendritic-cell maturation, antigen presentation, and costimulatory molecule expression, while inducing BAFF and APRIL production by myeloid cells and stromal cells. These signals support autoreactive B-cell survival, plasmablast differentiation, class-switch recombination, and autoantibody production. Thus, type I IFN may contribute to the initiation of autoimmunity by lowering the activation threshold of autoreactive lymphocytes in early disease, as well as to amplification of established inflammation by sustaining immune-complex formation, plasmablast expansion, and tissue recruitment of inflammatory cells [30].

IFN-I is implicated from the earliest stages of SLE, with IFN-II contributing to disease development and severity [33]. Plasmacytoid dendritic cells (pDCs) are the primary producers of IFN-I in vivo, generating large amounts and triggering an “antiviral” immune response, which explains the nonspecific symptoms of SLE such as fatigue, fever, myalgia, and arthralgia [34]. Elevated IFN-I levels and gene expression are observed in up to 50% of SLE patients and in affected organs such as the kidney in lupus nephritis (LN) [35]. Targeting IFN-I signaling—via agents like anifrolumab or JAK/TYK inhibitors—represents a promising therapeutic strategy to mitigate inflammation and prevent organ damage [36]. Therapeutic approaches include anifrolumab [37], a monoclonal antibody against the alpha IFN receptor (IFN-αR), and inhibitors of Janus kinases (JAKs) and tyrosine kinases (TYKs), as the receptor signaling pathway for IFN-α involves these proteins [38].

Neutrophils play a central role in systemic lupus erythematosus (SLE) by serving as a major source of autoantigens. As the most abundant circulating leukocytes, they act as first-line defenders against infection through several mechanisms, including the release of neutrophil extracellular traps (NETs), a regulated cell-death pathway. NETs consist of extracellular DNA and nuclear components, such as nucleosomes, decorated with antimicrobial proteins including neutrophil elastase, LL-37, HMGB1, proteinase 3, and myeloperoxidase [39,40,41,42]. Chromatin particles released by NETs are internalized by antigen-presenting cells (APCs), inducing type I interferon (IFN-I) production through TLR activation [35]. In SLE, neutrophil turnover is accelerated, leading to elevated apoptotic particles and mRNA in circulation, forming a “neutrophil signature” associated with anti-DNA antibody development and disease activity [43,44].

In SLE, NET release is promoted by type I interferons, immune complexes containing nucleic acids, anti-neutrophil and anti-DNA antibodies, complement activation, inflammatory cytokines, endothelial activation, oxidative stress, infections, and ultraviolet-induced tissue injury. Low-density granulocytes, which are expanded in some patients with active SLE, are particularly prone to spontaneous NET formation. By exposing DNA, histones, nucleosomes, LL-37, HMGB1, neutrophil elastase, myeloperoxidase, proteinase 3, and other modified nuclear or granular proteins, NETs protect nucleic acids from degradation, provide targets for autoantibodies, and amplify plasmacytoid dendritic-cell activation. NETs promote SLE by protecting nucleic acids from degradation and exposing modified nuclear autoantigens. Patients may generate autoantibodies against NET-associated DNA and peptides, enhancing autoimmunity and explaining ANCA positivity in some cases [45,46,47]. Neutrophils also respond strongly to IFN-I, endothelial activation, cytokines, autoantibodies, and nucleic acid-containing immune complexes, making them increasingly prone to NET release. This positions neutrophils as key contributors to both the initiation and amplification of inflammation in SLE [48].

## 5. Adaptive Immunity in SLE: From Tolerance Loss to Polyclonal Autoantibody Production

In systemic lupus erythematosus (SLE), there is continuous crosstalk between the innate and adaptive branches of the immune system. The adaptive response is characterized by the clonal expansion of autoreactive T and B lymphocytes, as well as long-lived plasma cells (PCs) [21].

Human B-cell development begins in the bone marrow, where mature naïve B cells are generated independently of the spleen [49]. These cells enter the bloodstream and secondary lymphoid organs, where antigen exposure and T cells help drive their differentiation into memory B cells or plasma cells (PCs), depending on cytokine signaling and antigenic stimulation. Plasma cells are effector B cells responsible for antibody production; after activation, many migrate back to the bone marrow, where specific niches support their long-term survival and enable sustained antibody secretion independent of antigen persistence (long-lived PCs) [50]. A fraction of PCs also resides in peripheral tissues—including spleen, mucosa, lymph nodes, and inflamed sites—where they are generally short-lived and require ongoing stimulation [50]. Memory B cells, unlike PCs, do not secrete antibodies but ensure a rapid secondary immune response after re-encounter with the same antigen. They undergo somatic hypermutation and class-switch recombination to optimize antibody affinity and preferentially localize to the splenic marginal zone and mucosal epithelium [51]. In contrast to short-lived plasmablasts and plasma cells, which usually survive for days to weeks, long-lived plasma cells may persist for years and potentially decades within specialized survival niches, particularly in the bone marrow. This longevity allows sustained autoantibody production even when the initiating antigenic stimulus is no longer detectable [50].

**Figure 2:**
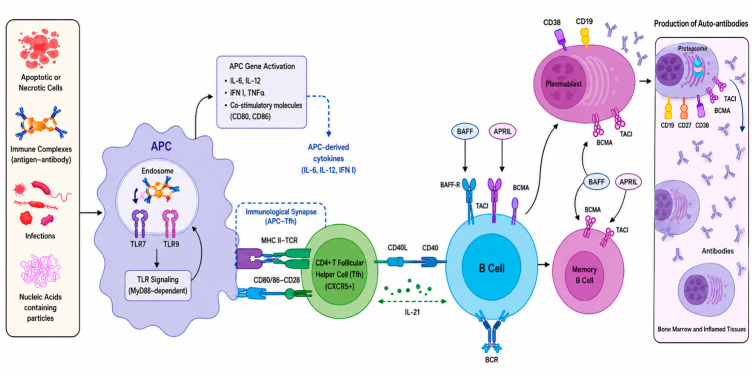
Mechanisms of B-cell activation in systemic lupus erythematosus. Nucleic acid-containing immune complexes are internalized by antigen-presenting cells through Fc receptors that bind the Fc portion of antibodies. Following internalization, DNA- or RNA-containing material activates endosomal Toll-like receptors, leading to APC activation, cytokine production, and increased expression of costimulatory molecules. Activated APCs present autoantigen-derived peptides to CD4+ T cells through MHC II–TCR interactions and provide additional costimulatory signals through CD80/86–CD28. CXCR5+ T follicular helper cells subsequently provide cognate B-cell help through CD40L–CD40 interactions and IL-21. B cells then differentiate into memory B cells, plasmablasts, and plasma cells, while survival niches in bone marrow and inflamed tissues sustain autoantibody production. Solid arrows indicate direct cellular events, receptor-mediated interactions, or differentiation pathways; dashed arrows indicate soluble cytokine-mediated signaling. Blue dashed arrows represent APC-derived cytokines, whereas green dashed arrows indicate Tfh-derived IL-21-mediated B-cell help.

Cytokines tightly regulate B-cell survival and differentiation, notably BAFF and APRIL, along with TNF-α, IL-6, and IL-21 [52]. BAFF and APRIL, produced by antigen-presenting cells activated by IFN-I and TNF-α, strongly promote B-cell proliferation and antibody secretion [52,53,54]. BAFF binds BAFF-R, TACI, and BCMA, while APRIL binds only TACI and BCMA. BAFF-R is essential for B-cell maturation, whereas TACI and BCMA are expressed on activated B cells and plasma cells [55,56,57]. Long-lived PCs express only TACI and BCMA, suggesting APRIL may be more relevant than BAFF for plasma cell survival [57]. Nonetheless, both cytokines support bone-marrow PC niches [58] (Figure 2).

Self-tolerance mechanisms normally eliminate autoreactive B cells, but failures in negative selection and dysregulated germinal-center reactions allow autoreactive clones to persist. Germinal-center dysregulation in SLE includes excessive T follicular helper-cell activity, insufficient regulatory T-cell and follicular regulatory T-cell control, increased BAFF and IL-21 signaling, abnormal somatic hypermutation and class-switch recombination, and defective deletion of autoreactive B-cell clones. These abnormalities favor the selection of high-affinity autoreactive B cells and their differentiation into memory B cells or plasma-cell precursors. In systemic lupus erythematosus (SLE), loss of tolerance occurs early, before B-cell activation. Endogenous nucleic acids drive chromatin-reactive B-cell proliferation through multiple pathways. First, APC-derived cytokines—including IFN-I, IL-6, TNF-α, and BAFF—promote maturation into memory B cells or PCs, germinal-center formation, and autoantibody production [52,57]. Second, nucleic acid-containing debris and immune complexes internalized via the BCR activate autoreactive B cells and induce expression of TACI, BAFF, and APRIL. Third, TLR-dependent activation (TLR7, TLR9) and other nucleic acid sensors such as MDA5 or cGAS contribute to T cell-independent B-cell activation [59]. Memory B-cells in SLE can also be activated through TLRs without T-cell help, promoting plasmablast expansion [24]. BCR engagement remains the main antigen-specific signal for B-cell activation, whereas TLR7 and TLR9 provide potent synergistic signals after BCR-mediated internalization of RNA- or DNA-containing antigens. This dual BCR–TLR activation is particularly efficient in SLE because nucleic-acid-containing immune complexes deliver both antigen specificity and innate danger signals. TLR stimulation alone may activate selected memory B-cell responses, but full autoreactive expansion is most efficient when BCR and TLR pathways cooperate.

Several B-cell subsets contribute to autoantibody production. Plasmablasts and short-lived and long-lived PCs directly secrete antibodies, while memory B cells act indirectly by generating new antibody-producing cells. Different autoantibody specificities correlate with distinct B-cell compartments: anti-dsDNA and anti-nucleosome antibodies—often associated with disease activity—are reduced by B-cell depletion, while anti-Sm, anti-Ro, and anti-La antibodies remain stable, suggesting production by long-lived PCs that lack CD20 and resist anti-CD20 therapies [60]. However, autoantibody production is not strictly compartmentalized, as the same specificity may arise from multiple subsets [61]. Long-lived PCs sustain chronic autoimmunity and are implicated in relapses. By producing antibodies targeting nucleic acid-containing self-antigens, they generate immune complexes that activate Fc and complement receptors, perpetuating inflammation. This supports clinical observations that patients with baseline anti-Ro, anti-Sm, or anti-U1RNP antibodies—likely produced by long-lived PCs—are at higher risk of early flare after B-cell depletion [62]. Beyond bone marrow niches, active SLE supports plasmablast and PC survival in secondary lymphoid tissues and inflamed organs, including kidneys in lupus nephritis. Some of these extramedullary PCs express plasma cell-associated markers such as CD138; however, CD138 is not specific for long-lived plasma cells and should not be interpreted as a marker of longevity per se. Long-lived plasma cells are better defined by their functional persistence, resistance to CD20-directed depletion, and dependence on specialized survival niches. As these cells lack CD20, they escape anti-CD20 therapy. Indirect elimination may occur by resolving inflammation, depriving PCs of survival niches, after which mature PCs—unable to migrate—gradually decline [63]. These and direct plasma cell-targeted strategies form part of therapeutic considerations discussed in subsequent sections.

T cells originate in the bone marrow and migrate to the thymus, where they mature into immunocompetent and self-tolerant naïve CD4+ T helper cells or CD8+ cytotoxic T cells through thymic repertoire selection [64]. After leaving the thymus, T cells continue to differentiate and circulate until activated by antigenic peptides presented by MHC molecules on antigen-presenting cells (APCs). While all nucleated cells can present antigens to CD8+ T cells, only dendritic cells, B cells, monocytes, and macrophages act as professional APCs capable of priming CD4+ T cells [65]. Activated T cells perform multiple functions: CD4+ subsets (Th1, Th2, Th17) orchestrate immune responses through cytokine secretion [65], promote B-cell maturation into memory cells and plasma cells, and activate cytotoxic T cells, neutrophils, and macrophages. CD8+ T cells exert direct cytotoxic activity, while regulatory CD4+ T cells (Tregs) suppress excessive immune responses and control autoreactive clones [66,67,68,69]. T cells play a central role in systemic lupus erythematosus (SLE), where several defects contribute to autoimmunity, including impaired negative selection of autoreactive T cells, abnormal T-cell receptor signaling, and reduced Treg function [70]. Among CD4+ T-cell subsets, CXCR5+ T follicular helper cells are particularly relevant because they localize to B-cell follicles and germinal centers, where they interact with B cells through CD40L–CD40, ICOS, and cytokines such as IL-21. These interactions promote germinal-center formation, somatic hypermutation, class-switch recombination, affinity maturation, memory B-cell generation, and plasma-cell differentiation. T-cell tolerance may be broken against peptides derived from nuclear and nucleic-acid-associated autoantigens, including nucleosomal and histone proteins, Sm and U1-RNP components, Ro/SSA, La/SSB, ribosomal P proteins, and other autoantigenic peptides presented by HLA class II molecules. These autoreactive CD4+ T cells can then provide help to B cells recognizing related nuclear antigens, facilitating epitope spreading and diversification of the autoantibody repertoire.

This T cell-dependent autoantibody generation amplifies inflammation through cytokines, B-cell activation, and accumulation of autoreactive plasma cells and memory B cells, sustaining SLE pathogenesis [71].

The loss of tolerance to self-antigens, leading to a polyclonal immune response, results in the hallmark diagnostic feature of systemic lupus erythematosus (SLE): the presence of autoantibodies, primarily directed against nuclear antigens (ANAs). Over one hundred autoantibodies have been identified in SLE, including not only ANA (such as anti-nucleosome, anti-dsDNA, anti-Sm, anti-Ro, anti-La, and anti-U1-RNP antibodies) but also antibodies against cytoplasmic antigens (e.g., certain ribosomal P proteins), cell membrane antigens, and phospholipid-associated antigens. However, it is important to note that not all these autoantibodies are pathogenic or specific to SLE; they may coexist with other conditions or be present without indicating systemic autoimmunity [72,73].

The pathogenic potential of autoantibodies depends on several factors, including antigen specificity, isotype and IgG subclass, affinity and avidity, glycosylation profile, ability to fix and complement, capacity to form circulating or in situ immune complexes, access to target tissues, Fc receptor engagement, and the local inflammatory microenvironment. Some autoantibodies mainly serve as diagnostic or classification markers, whereas others directly contribute to tissue injury by activating complement, engaging Fc receptors, promoting thrombosis, or depositing in target organs such as the kidney. This explains why systemic autoantibody positivity does not invariably translate into clinically overt organ damage.

## 6. Lupus Nephritis: Pathogenesis of Immune-Induced Renal Injury

Kidney involvement in systemic lupus erythematosus (SLE) appears in about 30% of patients at onset and rises to 50–60% within 10 years. Among those with lupus nephritis (LN), 10–30% progress to end-stage kidney disease after 20 years. LN adversely affects survival; infections and cardiovascular events are the leading causes of death, and their risk increases as chronic kidney disease (CKD) progresses. The development of LN is driven by systemic inflammation, as previously discussed, as well as intrinsic renal factors.

In this context, LN should not be regarded only as a renal complication of SLE, but as the renal expression of systemic immune dysregulation interacting with kidney-specific susceptibility factors. Circulating autoantibodies, complement activation, immune-complex localization, endothelial injury, resident renal-cell activation, and tubulointerstitial immune infiltration jointly determine whether systemic autoimmunity translates into clinically overt nephritis, histological class transition, chronic scarring, or progressive loss of kidney function.

**Figure 3:**
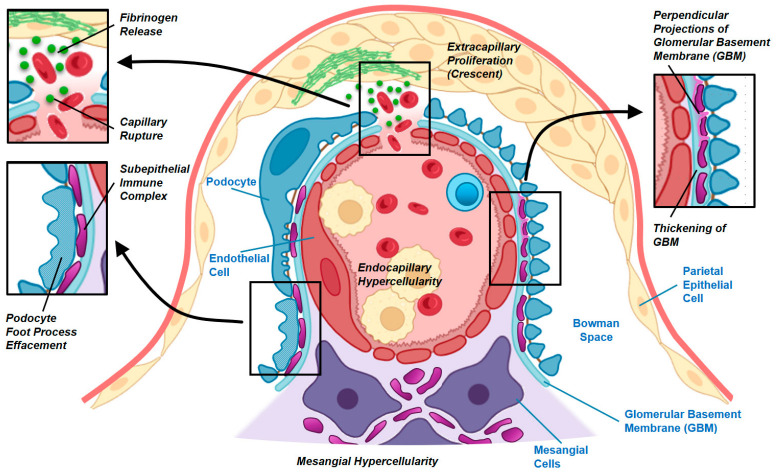
Renal histological lesions in lupus nephritis.

**Figure 4:**
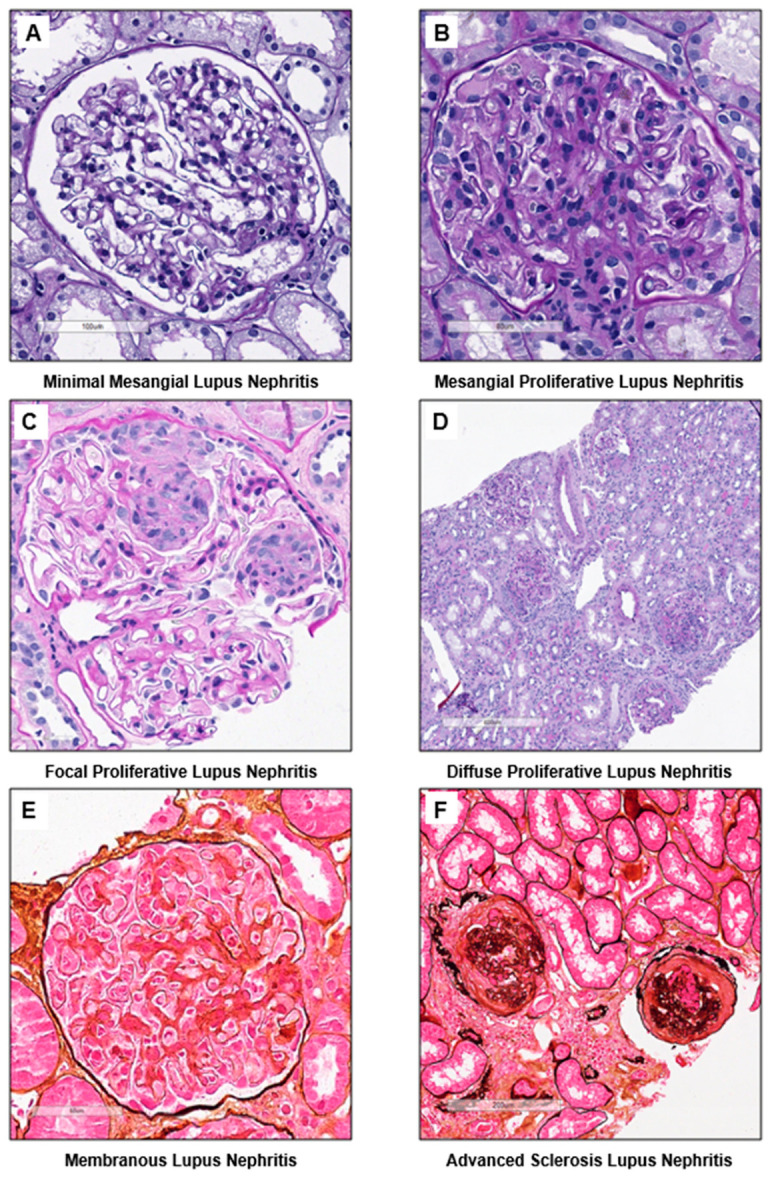
Renal histological patterns of lupus nephritis. (**A**) A normal glomerulus in class I lupus nephritis; (**B**) mesangial hypercellularity in class II lupus nephritis; (**C**) segmental endocapillary proliferation in a glomerulus from a patient with class III (focal proliferative lupus nephritis; (**D**) in diffuse proliferative LN > 50% of glomeruli displaying endo/extracapillary proliferation. (**E**) A glomerulus from class V lupus nephritis showing mild thickening of glomerular basement membranes and subepithelial deposits. (**F**) Diffuse global glomerulosclerosis in a patient with advanced class VI lupus nephritis. Histological images kindly provided by Prof. Michele Rossini (University of Bari).

Lupus nephritis (LN) develops through multiple converging pathogenic mechanisms, including: (a) direct deposition of nucleic acid-containing material in glomeruli, activating inflammation via Toll-like receptors (TLRs) on intrarenal macrophages, plasmacytoid dendritic cells (pDCs), mesangial cells, podocytes, and endothelial cells; (b) binding of polyclonal autoantibodies to nucleosomes and other autoantigens; (c) deposition of immune complexes (ICs) composed of DNA-containing nucleosomes formed in circulation or binding in situ to renal antigens [2,74,75]. The initial glomerular injury depends on where autoantibodies and ICs deposit. Circulating antibodies first localize to mesangial and/or subendothelial areas. Mesangial IC deposition induces mesangial hypercellularity and matrix expansion, leading to class II LN. Subendothelial deposits trigger endothelial activation, endocapillary hypercellularity, and recruitment of inflammatory cells, resulting in proliferative disease (classes III and IV). Capillary rupture releases fibrinogen, promoting proliferation of Bowman’s capsule parietal cells and formation of crescents. Subepithelial deposits produce the membranous pattern (class V), characterized by podocyte injury with foot process effacement. This pattern may occur with or without mesangial and subendothelial deposits. When present, basement membrane involvement reflects extension of earlier damage; when absent, in situ IC formation or antibody binding to renal antigens is more likely [76,77]. The former scenario yields mixed proliferative and non-proliferative lesions, whereas the latter presents as purely non-proliferative. Histological features vary with duration and intensity of IC exposure, and transitions between classes can occur. Current LN classifications are under revision, as the proliferative/non-proliferative distinction is considered imprecise [78]. Phase 2 of the ISN/RPS revision is expected to clarify these concepts [79]. A detailed discussion of the histologic classification of lupus nephritis is beyond the scope of this section; readers are referred to the original 2003 reports [80,81] and the 2018 revision [79]. However, the main renal histological lesions and examples of histological patterns of lupus nephritis are shown in Figure 3 and Figure 4, respectively.

Following immune complex (IC) deposition, complement activation occurs, contributing to cellular and tissue damage [82,83] and initiating an autoantigen-specific adaptive immune response within the kidney [2]. This response includes intrarenal production of cytokines such as interferon-alpha (IFN-I) [34,84,85], tumor necrosis factor-alpha (TNF-α), interleukin-17 (IL-17), and B-cell activating factor (BAFF). These mediators are secreted by renal resident cells (podocytes, endothelial cells, tubular epithelial cells, mesangial cells) [86] and by infiltrating immune cells, promoting recruitment of inflammatory cells into glomerular and interstitial compartments.

Infiltrating cells include macrophages, T and B lymphocytes, antigen-presenting cells (APCs) such as plasmacytoid (pDCs) and myeloid dendritic cells, and neutrophils. Immature myeloid and plasmacytoid dendritic cells infiltrate the tubulointerstitium in lupus nephritis, supporting persistent local inflammation [85].

Macrophages, present in both glomeruli and interstitium, derive from circulating monocytes or resident macrophages [87]. They exhibit diverse functions: pro-inflammatory M1 macrophages phagocytose pathogens, debris, and ICs, while M2 macrophages participate in tissue repair and remodeling [88]. Increased macrophage infiltration correlates with worse outcomes, including disease severity, progression, and future fibrosis or tubular atrophy. Mechanistically, macrophages may worsen renal injury by phagocytosing immune complexes, producing TNF-α, IL-1β, IL-6, reactive oxygen species, proteases, chemokines, and pro-fibrotic mediators such as TGF-β. These pathways sustain glomerular and tubulointerstitial inflammation, amplify leukocyte recruitment, promote tubular epithelial injury, and contribute to fibroblast activation, interstitial fibrosis, tubular atrophy, and irreversible nephron loss [89]. However, the specific prognostic roles of M1 versus M2 subsets remain incompletely defined [88].

Tubulointerstitial infiltrates contain dispersed B and T cells, occasionally forming aggregates and, rarely, tertiary lymphoid structures with germinal centers [87,90]. Activated T helper and cytotoxic T cells intensify inflammation [91], while B cells near T-cell aggregates differentiate into plasma cells (PCs). Circulating plasmablasts migrate into inflamed renal tissue and mature into short-lived plasma cells, which generally survive for days to weeks and usually less than one month. Persistent local antibody production in LN may therefore reflect continuous recruitment and differentiation of plasmablasts, as well as the presence of survival niches that support plasma-cell persistence [87,92]. The abundance of PCs correlates with histological activity and chronicity indices in LN [93,94]. Polyclonal activation of autoreactive B cells generates multiple autoantibodies, contributing to the classic ‘full-house’ immunofluorescence pattern of LN, characterized by simultaneous staining for IgG, IgA, IgM, C3, and C1q.

This localized autoantigen-driven immune response enhances recruitment and activation of additional APCs, including pDCs and myeloid dendritic cells, further amplifying adaptive immune pathways. Elevated dendritic cell infiltration is associated with fibrosis progression. Neutrophils contribute by releasing neutrophil extracellular traps (NETs) [39,95].

Damage to individual renal cells can trigger injury in others due to their integrated glomerular functions. Podocytes, endothelial cells, and mesangial cells interact structurally, and their activation or proliferation compromises glomerular integrity, leading to nephron loss [86,96]. Persistent inflammation in glomeruli and the tubulointerstitium drives glomerulosclerosis, interstitial fibrosis, and atrophy, which are irreversible changes associated with poorer outcomes [97,98]. In addition to ongoing inflammation, where infiltrating macrophages play a significant role [99,100,101], other factors such as persistent proteinuria and ischemia contribute to tubulointerstitial fibrosis [77,102,103,104]. Glomerular inflammation may worsen ischemia because the efferent arteriole supplies the peritubular capillaries; severe glomerulonephritis can therefore induce tubulointerstitial ischemia [105,106,107,108,109].

## 7. Therapeutic Strategies in Lupus Nephritis: A Histology-Oriented and Guideline-Based Approach

Treatment of LN is increasingly guided by the integration of histological class, activity and chronicity indices, renal function, degree of proteinuria, extrarenal SLE activity, comorbidities, fertility considerations, and previous treatment exposure. Contemporary management therefore combines general measures applicable to most patients—such as hydroxychloroquine, blood-pressure and proteinuria control, renin–angiotensin–aldosterone system blockade when appropriate, cardiovascular risk reduction, and glucocorticoid minimization—with class-specific immunosuppressive regimens and, in selected patients, biologic or targeted therapies [110].

The 2024 American College of Rheumatology guideline further reinforces this histology-oriented approach. It recommends prompt kidney biopsy when LN is suspected, particularly in patients with proteinuria > 0.5 g/g and/or otherwise unexplained impairment of kidney function. For active class III/IV LN, with or without class V, the guideline conditionally recommends triple immunosuppressive therapy based on glucocorticoids plus either mycophenolic acid analogs and belimumab, mycophenolic acid analogs and a calcineurin inhibitor, or low-dose Euro-Lupus cyclophosphamide followed by mycophenolic acid analogs plus belimumab. For pure class V LN with proteinuria ≥ 1 g/g, glucocorticoids plus mycophenolic acid analogs and a calcineurin inhibitor are recommended. The guideline also emphasizes lower-dose glucocorticoid regimens after intravenous pulses, early tapering, hydroxychloroquine use unless contraindicated, adjunctive nephroprotective measures, and close proteinuria monitoring [111].

Because the histological pattern of LN reflects the dominant mechanism of renal injury, therapeutic decisions should not rely solely on the presence of SLE-related kidney involvement but should integrate LN class, activity and chronicity indices, proteinuria burden, kidney function, extrarenal disease activity, and patient-specific factors. A histology-oriented therapeutic framework is summarized in Table 3.

Overall, this histology-oriented framework reflects the current shift from a traditional induction–maintenance paradigm toward an earlier, combination-based strategy aimed at rapid control of renal inflammation, reduction in glucocorticoid exposure, prevention of chronic kidney damage, and individualized long-term maintenance according to renal response and toxicity profile.

Traditional therapies for lupus nephritis (LN) have historically included antimalarials, glucocorticoids (GCs), and cytotoxic agents with broad immunomodulatory effects. More recently, targeted therapies have been introduced, and combination approaches integrating anti-inflammatory and immunosuppressive treatments with nephroprotective agents, such as renin–angiotensin–aldosterone system inhibitors and SGLT2 inhibitors, have become more common. Improved understanding of kidney repair dynamics and inflammation resolution, including mechanisms of immune complex reabsorption, may further refine treatment timing and strategies.

Anti-inflammatory therapies aim to control systemic SLE-related immune dysregulation, reducing acute renal injury and allowing intrinsic renal repair mechanisms to occur. Some treatments also exert kidney-specific effects, including GC pulses for acute inflammation, tacrolimus and voclosporin for podocyte injury, and complement inhibitors. Cyclophosphamide (CYC) additionally affects parietal epithelial cells, particularly in hyperplastic crescents [112]. An overview of current and emerging LN therapies is summarized in Table 4.

Given the importance of innate immune activation in SLE through TLR7 and TLR9, inhibition of these receptors and of type I interferon signaling is a key therapeutic strategy [113]. Hydroxychloroquine prevents endolysosomal acidification during antigen-presenting cell processing of nuclear particles and blocks endogenous nucleic-acid signaling through TLRs [114]. This dampens innate immune activation and reduces SLE activity, flare frequency, chronic organ damage—including CKD in LN—and improves survival [115,116]. Current guidelines recommend hydroxychloroquine for all SLE patients [117].

Rarely, antimalarials may cause toxic podocytopathy, leading to persistent or worsening proteinuria [118].

Glucocorticoids have been central to LN therapy since the mid-20th century, although long-term or high-dose GC exposure contributes substantially to infection, metabolic complications, cardiovascular risk, osteoporosis, and irreversible damage [117]. Intravenous methylprednisolone pulse therapy is generally reserved for active proliferative LN, clinically significant membranous LN, severe organ- or life-threatening SLE manifestations, and severe hematologic manifestations such as severe thrombocytopenia. Current recommendations support methylprednisolone pulses in the range of 250–1000 mg/day for 1–3 days, followed by lower-dose oral glucocorticoids and early tapering according to renal response, extrarenal activity, and toxicity risk [119]. This approach aims to preserve the rapid anti-inflammatory effects of glucocorticoids while minimizing cumulative exposure.

Cytotoxic agents suppress rapidly dividing immune cells. CYC and mycophenolate mofetil (MMF) are principal induction therapies, with maintenance typically involving low-dose GCs plus MMF or azathioprine (AZA) [117,120]. CYC can be administered using the NIH regimen, the Euro-Lupus regimen—which reduces cumulative dose while maintaining efficacy—or orally, though the latter requires higher doses and carries more toxicity [52]. CYC disrupts DNA replication, targeting proliferating T and B lymphocytes. MMF selectively inhibits inosine-5′-monophosphate dehydrogenase, blocking de novo purine synthesis in lymphocytes. MMF is at least as effective as CYC for induction [120] and may offer particular benefit in African American patients [121]. Notably, its active metabolite also exerts anti-inflammatory and anti-fibrotic effects [122].

Calcineurin inhibitors (CNIs)—cyclosporine (CsA), tacrolimus, and voclosporin—have demonstrated efficacy in LN. All inhibit calcineurin, thereby suppressing IL-2 transcription and T-cell activation [123,124], while also reducing autoantibody production indirectly [52]. CNIs additionally exert antiproteinuric effects through podocyte stabilization [125]. CsA reduces proteinuria and induces remission [126,127,128], though relapses, hypertension, and nephrotoxicity limit use. Tacrolimus is more potent and is effective in combination with MMF, although regional variation exists [129,130]. Voclosporin, a structurally modified CsA analog, was recently approved as an add-on therapy in LN, showing improved potency, favorable tolerability, and minimal drug interaction with MMF [131,132].

AZA inhibits DNA replication and purine synthesis. Although widely used in maintenance therapy, MMF has shown superior efficacy in many studies, restricting AZA use to MMF intolerance [121]. However, the MAINTAIN trial’s long-term follow-up showed no superiority of MMF over AZA in a European cohort [133]. Higher doses of AZA may be needed to inhibit humoral response and cellular activity [134].

Targeted therapies for LN, mainly biologics, focus on specific aberrant pathways or cellular targets. Current and emerging strategies primarily address B cells, plasma cells, T-cell/B-cell costimulation, and complement-mediated injury.

Therapeutic strategies targeted to B cells include B-cell depletion (rituximab, obinutuzumab), inhibition of B-cell survival factors (belimumab, atacicept), disruption of B-cell/T-cell interactions (abatacept), and plasma cell targeting (bortezomib, daratumumab) [135].

Belimumab, an anti-BAFF monoclonal antibody, is the first biologic approved specifically for LN. The BLISS-LN trial showed improved renal outcomes, especially in proliferative LN, with good tolerability [136]. Current evidence suggests that belimumab may also reduce the risk of LN flare-ups and decline in estimated glomerular filtration rate (eGFR) [137]. Belimumab markedly reduces naïve and transitional B cells but has limited effects on memory B cells and plasma cells, which are less BAFF-dependent [138,139,140,141]. However, medium- to long-term treatment may reduce plasma cells and autoantibody levels.

Rituximab, an anti-CD20 antibody, induces ADCC and complement-mediated cytotoxicity, but trials such as EXPLORER and LUNAR failed to meet primary endpoints in SLE and LN [142,143], likely because plasmablasts and plasma cells lack CD20 expression [61]. High-dose GCs and limited rituximab exposure may have contributed to trial limitations [144]. Rituximab increases BAFF levels following B-cell depletion, potentially enabling autoreactive B-cell reconstitution [145]. The CALIBRATE trial testing rituximab + CYC followed by belimumab showed improved immunologic responses but not superior clinical efficacy [146]. Despite mixed trial results, rituximab is effective in many observational LN cohorts, though it insufficiently targets long-lived plasma cells.

Agents such as bortezomib (proteasome inhibitor) and daratumumab (anti-CD38) target both short- and long-lived plasma cells, offering promise for refractory LN, though safety concerns regarding infection remain [147,148]. Daratumumab is currently being evaluated in active LN in a phase 2 open-label trial (NCT04868838). Caution is warranted due to the risk of infections associated with nonspecific depletion of long-lived plasma cells that produce protective IgG.

To address some limitations of rituximab, obinutuzumab, a glycoengineered type II anti-CD20 monoclonal antibody with enhanced antibody-dependent cellular cytotoxicity, demonstrated improved renal responses in the phase II NOBILITY trial and significantly higher complete renal response rates when added to standard therapy in the phase III REGENCY trial. Based on these findings, obinutuzumab was approved by the US Food and Drug Administration in October 2025 for the treatment of adult patients with active lupus nephritis receiving standard therapy [149,150,151].

CD19-targeting therapies, including tafasitamab, blinatumomab, or CD19 CAR-T cells, may better target plasmablasts and subsets of plasma cells due to broader CD19 expression, which is increased in SLE [152].

Additionally, abatacept, a fusion protein combining the Fc region of IgG1 with the extracellular domain of CTLA-4, intervenes in B-cell and T-cell interactions by blocking the CD28-CD80/86 costimulatory signal. Clinical trials with abatacept have shown a reduction in proteinuria in proliferative LN and decreased biological activity, though it did not achieve complete renal response over 52 weeks [52,152,153].

Complement activation contributes to immune complex-mediated injury but plays essential roles in apoptotic cell clearance, making complement inhibition a double-edged sword [154,155]. Current research focuses on terminal complement blockade, aiming to preserve early components while preventing downstream inflammatory damage [156]. Eculizumab, an anti-C5 monoclonal antibody, has limited evidence in LN but has been used in refractory antiphospholipid syndrome with thrombotic microangiopathy [157,158]. Ravulizumab, a longer-acting anti-C5 monoclonal antibody, is currently being evaluated in proliferative LN in the SANCTUARY trial (NCT04564339).

Current therapies poorly target long-lived plasma cells, which are central to persistent autoantibody production. Although inflammation reduction can diminish survival niches for these cells in affected tissues, long-lived bone marrow plasma cells remain challenging to eradicate [50,159]. Complete immunoablation or bone marrow transplantation can eliminate them but is impractical for most patients [160,161]. More selective strategies remain under investigation [58]. Other investigational therapies include agents targeting plasmacytoid dendritic cells (daxcilimab, litifilimab), interleukins (secukinumab, ustekinumab), and JAK/TYK2 pathways [20,162].

Another innovative strategy is chimeric antigen receptor (CAR) T-cell therapy, based on the genetic modification of T cells to recognize and target specific antigens, such as CD19. This approach, particularly anti-CD19 CAR T cells, represents a promising frontier. These engineered T cells can migrate to inflamed tissues and deplete B cells and plasmablasts [163]. Early experience in LN is limited but promising [164]. Effectiveness may be reduced when autoimmunity is driven by CD19-negative long-lived plasma cells, but CAR-T approaches remain a major step toward personalized immunomodulation [164].

## 8. Conclusions

The understanding of systemic lupus erythematosus (SLE) pathogenesis has significantly advanced over time. It can now be characterized by a breakdown in self-tolerance prior to the activation of B cells, which subsequently leads to an inappropriate adaptive immune response. Despite this progress, many molecular pathways involved in SLE remain complex and are not always easily discernible with current clinical tools. Identifying distinct phenotypic subgroups associated with specific pathological mechanisms is crucial for the development of personalized treatment strategies, as demonstrated in other rheumatic diseases such as rheumatoid arthritis (RA) [165]. Although RA has seen significant progress in understanding its pathogenesis and the introduction of targeted therapies, personalized treatment approaches are still evolving and have yet to substantially alter clinical management. Similarly, lupus nephritis (LN) may encounter comparable challenges. A promising direction for future research is the molecular stratification of renal tissue injury patterns, which is likely to be pivotal for advancing treatment strategies in the coming years.

## Figures and Tables

**Table 1:** Summary of SLE classification criteria and weight assignments (ACR/EULAR 2019).

Clinical Domains and Criteria	Weight
*Constitutional*	
Fever: temperature ≥ 38·3 °C	2
*Hematological*	
Leucopenia: <4 × 10^9^ per L	3
Thrombocytopenia: <100 × 10^9^ per L	4
Autoimmune hemolysis: positive direct Coomb’s test, and evidence of hemolysis (reticulocytosis, low haptoglobin, and elevated indirect bilirubin or lactate dehydrogenase)	4
*Neuropsychiatric*	
Delirium	
Psychosis: characterized by delusions, hallucination, or both, in the absence of delirium	3
Seizure: primary generalized or partial or focal seizure	5
*Mucocutaneous*	
Non-scarring alopecia	2
Oral ulcers	2
Subacute cutaneous or discoid lupus (clinical or biopsy-proven)	4
Acute cutaneous lupus: localized form is the classic fixed, flat, or raised erythema over malar eminences, but tends to spare the nasolabial fold; can be generalized in distribution	6
*Musculoskeletal*	
Joint involvement: inflammatory joint pain characterized by synovitis involving two or more joints, or tenderness in two or more joints and morning stiffness	6
*Renal*	
Proteinuria: >0.5 g/24 h or equivalent spot urine protein–creatinine ratio	4
Renal biopsy class II or V lupus nephritis	8
Renal biopsy class III or IV lupus nephritis	10
**Immunologic Domains and Criteria**	**Weight**
*Antiphospholipid antibodies*	
At least moderate-titer anti-cardiolipin antibodies (any isotype), or positive anti-β2- glycoprotein 1 antibodies or lupus anticoagulant	2
*Complement Proteins*	
Low C3 or low C4	3
Low C3 and low C4	4
*SLE-specific antibodies*	
Anti-double-stranded DNA antibody in an immunoassay with demonstrated 90% or greater specificity for SLE against relevant disease controls or anti-Sm antibody	6

According to the 2019 EULAR/ACR SLE classification criteria, an ANA titer ≥ 1:80 on HEp-2 cells (ever) is the mandatory entry requirement. Once this is met, classification relies on weighted clinical and immunological items grouped by domains. Attribution is essential: an item must not be counted if a more plausible non-SLE explanation exists. Occurrence on at least one episode is sufficient. For SLE classification, patients must accumulate ≥ 10 points and include at least one clinical criterion. To avoid overweighting, only the highest-scoring item within a given domain contributes to the total; additional items in the same domain are ignored. The framework clarifies the spectrum of disease manifestations and supports consistent patient classification across studies and practice, without replacing clinical diagnosis and judgment. SLE: systemic lupus erythematosus.

**Table 2:** Different SLE-related autoantibodies seen at onset [4].

Autoantibodies	Approximate Frequency *	Main Clinical/Diagnostic Associations
ANA ^§^	95–100%	Highly sensitive screening marker; absence strongly argues against SLE, but rare ANA-negative SLE has been reported
Anti–double-stranded DNA	60–80%	High specificity; associated with disease activity, complement consumption, lupus nephritis, and flare risk
Anti–nucleosome	60–70%	Associated with immune-complex formation, disease activity, and renal involvement
Anti–histone	60–70%	Common in SLE; strongly associated with drug-induced lupus but may occur in idiopathic SLE
Anti–Ro/SSA	30–40%	Photosensitive cutaneous disease, subacute cutaneous lupus, neonatal lupus, congenital heart block, sicca/overlap features
Anti–La/SSB	5–10%	Often associated with anti-Ro/SSA; sicca features, neonatal lupus risk
Anti–(U1)RNP	15–30%	Raynaud phenomenon, overlap connective-tissue disease features, mixed connective-tissue disease phenotype
Anti–Sm	10–30%	Highly specific for SLE; supports diagnosis even when clinical features are heterogeneous
Anti–Ribosomal P	5–15%	Reported association with neuropsychiatric SLE and hepatic involvement in some cohorts
Anti–Ku	5–10%	Overlap syndromes, myositis/scleroderma-like features
Anti–PCNA	1–5%	Rare but relatively specific; limited clinical use
Anti–β2-glycoprotein I	30–45%	Antiphospholipid syndrome, thrombosis, pregnancy morbidity
Anti–cardiolipin (aCL)	30–70%	Antiphospholipid syndrome, thrombosis, pregnancy morbidity

* Frequencies vary across cohorts, ethnicity, disease duration, and assay methodology. ANA: antinuclear antibodies; SLE: systemic lupus erythematosus; PCNA: proliferating cell nuclear antigen. ^§^ Lack of ANA is uncommon in SLE and strongly argues against this diagnosis; ANA positivity usually precedes clinical disease onset by several years.

**Table 3:** Histology-oriented therapeutic strategies in lupus nephritis according to renal histology and clinical setting [111].

LN Class/ Clinical Setting	Main Pathological Feature	Treatment Goal	Preferred Initial Therapeutic Approach	Maintenance/ Follow-Up Considerations
**Class I—Minimal mesangial LN**	Normal glomeruli by light microscopy with mesangial immune deposits detectable by immunofluorescence and/or electron microscopy	Control extrarenal SLE activity and monitor for renal evolution	No kidney-specific immunosuppression is usually required. Treat according to extrarenal SLE manifestations; hydroxychloroquine is generally recommended unless contraindicated.	Periodic monitoring of urinalysis, proteinuria, serum creatinine/eGFR, complement levels, and anti-dsDNA antibodies. Repeat renal evaluation if proteinuria, hematuria, or kidney dysfunction develops.
**Class II—Mesangial proliferative LN**	Mesangial hypercellularity and/or mesangial matrix expansion with immune deposits limited mainly to the mesangium	Control mild urinary abnormalities and prevent progression	Hydroxychloroquine and optimized supportive care. Low-dose glucocorticoids may be considered when renal manifestations coexist with active extrarenal disease. Significant proteinuria should prompt careful reassessment for podocytopathy or transformation to a higher class.	Monitor proteinuria and kidney function. Persistent or increasing proteinuria, active urinary sediment, or declining eGFR should prompt consideration of repeat biopsy.
**Class III—Focal proliferative LN**	Active or chronic proliferative lesions involving < 50% of glomeruli, often with subendothelial immune deposits and inflammatory cell infiltration	Rapid suppression of active inflammation, preservation of nephron mass, and prevention of chronic scarring	Prompt glucocorticoid therapy, usually with intravenous methylprednisolone pulses followed by a lower-dose oral glucocorticoid regimen and taper. In line with the 2024 ACR guideline, active class III/IV LN should preferably be treated with a triple immunosuppressive regimen including glucocorticoids plus: (i) mycophenolic acid analog plus belimumab; or (ii) mycophenolic acid analog plus a calcineurin inhibitor; or (iii) low-dose Euro-Lupus cyclophosphamide plus belimumab, followed by mycophenolic acid analog after completion of cyclophosphamide.	Maintenance therapy generally includes MMF or azathioprine with glucocorticoid tapering. Monitor renal response, proteinuria reduction, complement/anti-dsDNA trends, treatment toxicity, and adherence.
**Class IV—Diffuse proliferative LN**	Active or chronic proliferative lesions involving ≥ 50% of glomeruli; often associated with endocapillary proliferation, necrosis, crescents, and high inflammatory burden	Achieve rapid disease control, prevent irreversible chronic damage, and reduce risk of kidney failure		Long-term maintenance is required. Taper glucocorticoids as early as clinically feasible. Close monitoring is needed for incomplete response, relapse, chronicity progression, infection risk, gonadal toxicity with cyclophosphamide, and drug-specific adverse effects.
**Mixed Class III/IV + V LN**	Proliferative LN combined with membranous lesions and subepithelial immune deposits; frequently associated with substantial proteinuria	Control proliferative inflammation while reducing proteinuria and podocyte injury	Treat primarily as proliferative LN. A triple immunosuppressive regimen should be considered according to the active proliferative component. Calcineurin inhibitor-containing regimens may be particularly useful when nephrotic-range proteinuria or podocyte injury is prominent.	Maintenance should address both inflammatory activity and persistent proteinuria. Continue antiproteinuric supportive therapy and monitor for relapse, chronic damage, and CNI nephrotoxicity when CNIs are used.
**Class V—Pure membranous LN**	Diffuse subepithelial immune deposits with podocyte injury and glomerular basement membrane remodeling, without active proliferative lesions	Reduce proteinuria, prevent nephrotic complications, and preserve kidney function	Supportive and nephroprotective therapy is central in all patients, including hydroxychloroquine unless contraindicated, blood-pressure control, RAAS blockade when appropriate, and management of cardiovascular and thrombotic risk. In line with the 2024 ACR guideline, pure class V LN with proteinuria ≥ 1 g/g should be treated with a triple regimen including glucocorticoids, mycophenolic acid analog, and a calcineurin inhibitor. For proteinuria < 1 g/g, treatment should be individualized and may include glucocorticoids and/or immunosuppressive therapy according to renal and extrarenal disease activity.	Follow proteinuria trajectory closely. Consider mycophenolate mofetil-based regimens, CNIs, or biologic add-on therapy in selected cases. Monitor for edema, hypoalbuminemia, thrombosis risk, and treatment toxicity.
**Class VI—Advanced sclerosing LN**	Global sclerosis involving most glomeruli, reflecting advanced irreversible chronic damage	Avoid unnecessary immunosuppression and manage chronic kidney disease	Kidney-directed immunosuppression is usually not beneficial unless there is evidence of active extrarenal SLE or residual active renal inflammation. Focus on CKD care, blood-pressure control, proteinuria reduction, cardiovascular risk management, and preparation for kidney replacement therapy when needed.	Monitor CKD progression, complications of reduced kidney function, cardiovascular risk, anemia, bone-mineral metabolism, and transplant eligibility. Immunosuppression should be individualized and minimized when active inflammatory disease is absent.
**Refractory or relapsing LN**	Persistent active lesions, incomplete renal response, recurrent flares, or progression despite appropriate induction/maintenance therapy	Reassess diagnosis, distinguish activity from chronicity, and identify treatable mechanisms	In patients with inadequate response, the first step should be reassessment of adherence, drug dosing, drug exposure, and alternative causes of kidney injury. If response remains inadequate after appropriate therapy, escalation from dual to triple therapy, switching to an alternative triple regimen, addition of anti-CD20 therapy, or referral for investigational therapy should be considered. Repeat kidney biopsy should be considered when clinical findings are discordant with the expected response or when activity versus chronicity is unclear.	Long-term strategy should be guided by response kinetics, histological activity/chronicity, cumulative toxicity, infection risk, fertility considerations, and patient preferences. Multidisciplinary follow-up is recommended.

anti-dsDNA: anti-double-stranded DNA antibodies; CKD: chronic kidney disease; CNI: calcineurin inhibitor; eGFR: estimated glomerular filtration rate; LN: lupus nephritis; MMF: mycophenolate mofetil; SLE: systemic lupus erythematosus; RAAS, renin–angiotensin–aldosterone system.

**Table 4:** Overview of current and emerging treatments for lupus nephritis.

Therapy Type	Agents	Mechanism of Action	Notes/Benefits	Limitations/Side Effects
**Conventional Therapies**	Hydroxychloroquine	Inhibits TLR signaling and endolysosomal acidification	Reduces SLE activity and flares; improves survival	Rare podocytopathy
	Glucocorticoids (e.g., methylprednisolone)	Anti-inflammatory and immunosuppressive	Rapid inflammation control; GC-sparing strategies reduce side effects	Long-term use linked to comorbidities
	Cyclophosphamide (CYC)	DNA replication inhibition	Effective in induction therapy	Cytotoxicity; regimen-dependent side effects
	Mycophenolate mofetil (MMF)	Inhibits purine synthesis in B/T cells	Comparable to CYC; better tolerated in some populations	GI side effects; teratogenic
	Azathioprine (AZA)	Purine analog	Used in maintenance when MMF not tolerated	Less effective than MMF in some cohorts
**Calcineurin Inhibitors (CNIs)**	Cyclosporine (CsA), Tacrolimus, Voclosporin	Inhibit IL-2 transcription; podocyte protection	Reduce proteinuria; voclosporin approved for LN	Nephrotoxicity, hypertension
**Biologics—B cell Targeted**	Rituximab	Anti-CD20; B-cell depletion via ADCC/CDC	Effective in observational studies	Limited effect on plasma cells; trial failures
	Belimumab	Anti-BAFF; reduces naïve B cells	Approved for LN; reduces flares	Slow onset; limited plasma cell impact
	Obinutuzumab	Enhanced anti-CD20 activity	Improved complete renal response when added to standard therapy in the phase III REGENCY trial	FDA-approved for active LN in adults receiving standard therapy; infusion-related reactions and infection risk require monitoring
	Daratumumab, Bortezomib	Target plasma cells (CD38, proteasome)	Used in refractory cases	Risk of infections; repopulation of PCs
**T-** **cell** **/B-cell Interaction Blockers**	Abatacept	Blocks CD28-CD80/86 costimulation	Reduces proteinuria	Incomplete renal response in trials
**Complement** **Inhibitors**	Ravulizumab	Anti-C5; prevents terminal complement activation	Preserves early complement functions	Under investigation; infection risk
**Emerging Therapies**	CAR T-cell therapy (anti-CD19)	Engineered T cells targeting B cells	Potential for deep depletion of autoreactive cells	Limited data; may not target CD19– PCs
	Anti-interleukin agents (e.g., secukinumab)	Target specific cytokines	Personalized immunomodulation	Still experimental

ADCC: Antibody-dependent cellular cytotoxicity; BAFF: B-cell activating factor; CAR-T: Chimeric antigen receptor T; CDC: Complement-dependent cytotoxicity; IFN-I: Type I interferon; TLR: Toll-like receptor.

## Data Availability

No new data were created or analyzed in this study. Data sharing is not applicable to this article.

## Abbreviations

The following abbreviations are used in this manuscript:

ADCC	Antibody-Dependent Cellular Cytotoxicity
ANA	Antinuclear Antibodies
ANCA	Anti-Neutrophil Cytoplasmic Antibodies
APC	Antigen-Presenting Cell
APRIL	A Proliferation-Inducing Ligand
AZA	Azathioprine
BAFF	B-cell Activating Factor
BAFF-R	BAFF Receptor
BCMA	B-cell Maturation Antigen
BCR	B-cell Receptor
CAR-T	Chimeric Antigen Receptor T cells
CDC	Complement-Dependent Cytotoxicity
CKD	Chronic Kidney Disease
CNI	Calcineurin Inhibitor
CsA	Cyclosporine A
CYC	Cyclophosphamide
DC	Dendritic Cell
DNases	Deoxyribonucleases
dsDNA	Double-Stranded DNA
eGFR	Estimated Glomerular Filtration Rate
EMA	European Medicines Agency
FDA	U.S. Food and Drug Administration
GC	Glucocorticoid(s)
HEp-2	Human Epithelial Type-2 (cell line)
HLA	Human Leukocyte Antigen
HMGB1	High-Mobility Group Box 1
IC	Immune Complex
IFN-I	Type I Interferon
IFNAR (IFN-αR)	Type I Interferon Receptor
ISN/RPS	International Society of Nephrology/Renal Pathology Society
JAK	Janus Kinase
LN	Lupus Nephritis
MDA5	Melanoma Differentiation-Associated Protein 5
MHC	Major Histocompatibility Complex
MMF	Mycophenolate Mofetil
NETs	Neutrophil Extracellular Traps
NIH	National Institutes of Health
NK	Natural Killer (cell)
PC	Plasma Cell
PCNA	Proliferating Cell Nuclear Antigen
pDC	Plasmacytoid Dendritic Cell
RA	Rheumatoid Arthritis
RAAS	Renin–Angiotensin–Aldosterone System
RNP	Ribonucleoprotein (e.g., U1-RNP)
SGLT2	Sodium–Glucose Cotransporter-2
SLE	Systemic Lupus Erythematosus
STAT	Signal Transducer and Activator of Transcription
TACI	Transmembrane Activator and CAML Interactor
TCR	T-Cell Receptor
Tfh	Follicular Helper T cell
Th	T helper cell
TLR	Toll-Like Receptor
TNF	Tumor Necrosis Factor
TNF-α	Tumor Necrosis Factor-Alpha
Tregs	Regulatory T cells
TYK/TYK2	Tyrosine Kinase/Tyrosine Kinase 2
U1-RNP	U1-Ribonucleoprotein
UCTD	Undifferentiated Connective-Tissue Disease
UV	Ultraviolet
aβ2GPI	Anti-β2-Glycoprotein I Antibodies
aCL	Anti-Cardiolipin Antibodies
